# Tracing the subducting Pacific slab to the mantle transition zone with hydrogen isotopes

**DOI:** 10.1038/s41598-021-98307-y

**Published:** 2021-09-21

**Authors:** Takeshi Kuritani, Kenji Shimizu, Takayuki Ushikubo, Qun-Ke Xia, Jia Liu, Mitsuhiro Nakagawa, Hajime Taniuchi, Eiichi Sato, Nobuo Doi

**Affiliations:** 1 Department of Earth and Planetary Sciences, Faculty of Science, Hokkaido University, Sapporo, Japan; 2 Kochi Institute for Core Sample Research, Japan Agency for Marine-Earth Science and Technology, Nankoku, Japan; 3 Key Laboratory of Geoscience Big Data and Deep Resource of Zhejiang Province, School of Earth Sciences, Zhejiang University, Hangzhou, China; 4 Department of Natural History Sciences, Graduate School of Science, Hokkaido University, Sapporo, Japan; 5 Earth Science Laboratory, Hokkaido University of Education, Asahikawa, Japan; 6 Research Center for Regional Disaster Management, Iwate University, Morioka, Japan

**Keywords:** Planetary science, Solid Earth sciences

## Abstract

Hydrogen isotopes have been widely used as powerful tracers to understand the origin of terrestrial water and the water circulation between the surface and the deep interior of the Earth. However, further quantitative understanding is hindered due to a lack of observations about the changes in D/H ratios of a slab during subduction. Here, we report hydrogen isotope data of olivine-hosted melt inclusions from active volcanoes with variable depths (90‒550 km) to the subducting Pacific slab. The results show that the D/H ratio of the slab fluid at the volcanic front is lower than that of the slab fluid just behind the volcanic front. This demonstrates that fluids with different D/H ratios were released from the crust and the underlying peridotite portions of the slab around the volcanic front. The results also show that the D/H ratios of slab fluids do not change significantly with slab depths from 300 to 550 km, which demonstrates that slab dehydration did not occur significantly beyond the arc. Our estimated δD‰ value for the slab materials that accumulated in the mantle transition zone is > − 90‰, a value which is significantly higher than previous estimates.

## Introduction

Global water cycles involving the Earth’s deep interior have played a critical role in Earth’s evolution^1–5^. Water in the Earth’s mantle affects its rheological and melting behaviours, thereby controlling the thermal evolution and chemical differentiation of the solid Earth^2^. The ocean’s mass is primarily controlled by the balance between the water influx to the interior through subducting oceanic plates and the water outflux to the surface through magmatism^3–5^. Understanding the origin of terrestrial water and the cycling and distribution of water in the Earth’s interior has been significantly improved by the application of stable hydrogen isotopes, which are powerful tracers^6–13^. As materials transported into the Earth’s interior by subduction decrease the hydrogen isotope (D/H) ratios by releasing Deuterium (D)-enriched fluids^9^, D/H ratios of deep-source magmas allow us to identify the involvement of the recycled water as well as distinguish the recycled water from the Earth’s primordial water.

Little is understood about the changes in the D/H ratio of subducting slabs with increasing depth, which is a key issue in estimating the characteristic D/H ratios of subducted recycled materials in the deep mantle reservoir. Using the results of hydrogen isotopic analyses on olivine-hosted melt inclusions for some Mariana arc volcanoes, Shaw et al.^9^ estimated the δD‰ values (δD‰ = [(D/H)_Sample_ − (D/H)_SMOW_]/(D/H)_SMOW_ × 1000, where SMOW refers to the standard mean ocean water, and δD = 0‰) of the subducted materials after 92% dehydration^14^ to be as low as − 234‰. Walowski et al.^11^ estimated the δD‰ values of dehydrated residual slab materials to be − 100‰ to − 120‰ by combining the results of hydrogen isotopic analyses on melt inclusions from Cascadia arc basalts with a model that considered the thermal and petrological structures of a hot subducting slab. So far, however, the evolution of the D/H ratios of the subducting materials beyond the frontal arc has not been well defined by natural observations. This is partly because the density of volcanoes decreases from the volcanic front to the rear-arc side, and the number of rear-arc volcanoes is relatively small (subduction zone volcanoes with slab depths (*Z*) of > 150 km are only ~ 7%)^15^. In addition, subduction zone magmas typically contain appreciable amounts of volatiles, and, resultingly, they commonly degas during their ascent to the surface. Therefore, to estimate the D/H ratios of magma at depths using subaerial volcanic products, it is necessary to analyse melt inclusions (typically < 100 μm in diameter) trapped in phenocrysts^9,11^. However, it is challenging to obtain high-quality hydrogen isotopic data on samples of such small sizes. Furthermore, D/H data of the melt inclusions should be used carefully, because the ratios do not necessarily represent those of the original magma due to degassing and post-entrapment modification of the melt inclusion compositions by shallow-level processes^9,11,16,17^.

**Figure 1:**
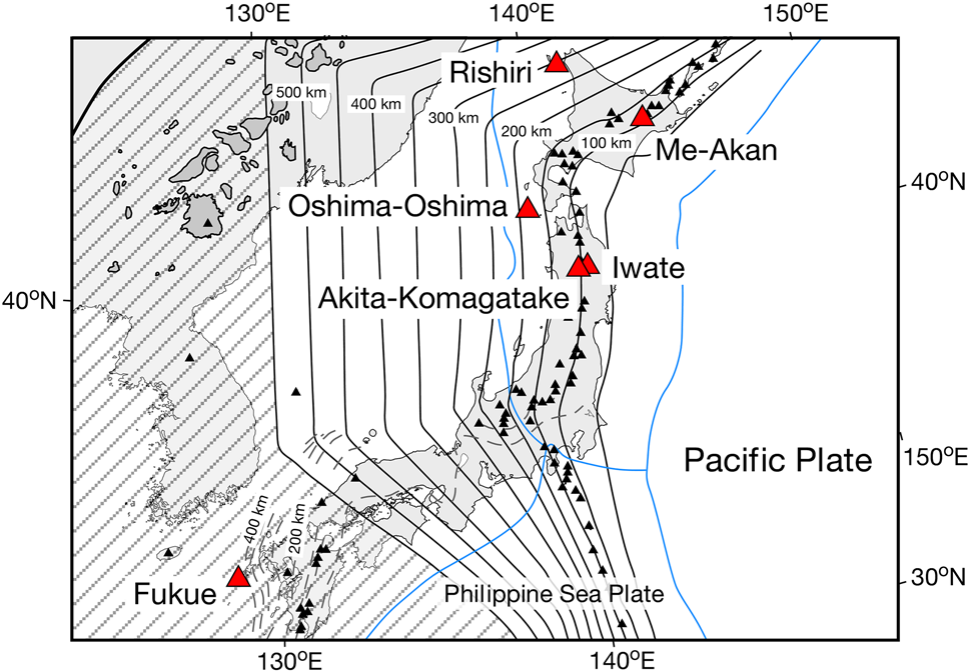
Map showing the distribution of active volcanoes above the subducted Pacific slab. The studied active volcanoes are shown here: Iwate (*Z* =  ~ 90 km), Akita-Komagatake (*Z* = ~ 100 km), Me-Akan (*Z* = ~ 110 km), Oshima-Oshima (*Z* = ~ 180 km), Rishiri (*Z* = ~ 300 km), and Fukue (*Z* = ~ 550 km). The slab depths were sourced from Refs.^38,39^. The grey patches denote representative Cenozoic volcanic fields in China^40^. The solid and dashed contour lines denote the depths of the upper boundaries of the subducting Pacific and Philippine Sea slabs, respectively^39^. The blue lines denote the plate boundaries at the surface. The shaded area indicates the range of the stagnant Pacific slab at depths > ~ 550 km in the MTZ^41^. This figure was created using Canvas 14.

In this study, to trace the evolution of the D/H ratios of the subducting Pacific slab from the volcanic front to the mantle transition zone (MTZ), the hydrogen isotopic compositions of olivine-hosted melt inclusions were determined for basaltic scoria samples from six active volcanoes, including Iwate (*Z* = ~ 90 km), Akita-Komagatake (*Z* = ~ 100 km), Me-Akan (*Z* = ~ 110 km), Oshima-Oshima (*Z* = ~ 180 km), Rishiri (*Z* = ~ 300 km), and Fukue (*Z* = ~ 550 km) (Fig. 1). The Iwate, Akita-Komagatake, and Oshima-Oshima volcanoes belong to the NE Japan arc, and the Me-Akan and Rishiri volcanoes belong to the Kuril arc. Fukue is an intraplate-type volcano, beneath which the subducted Pacific Plate is stagnated in the MTZ (Fig. 1). The MTZ here is considerably hydrous, as shown by electrical conductivity observations^18^. The influx of fluids derived from the stagnant Pacific slab generates primary magmas from the melting of the asthenospheric mantle at the Fukue volcano^19^. Therefore, the samples from the five arc volcanoes and the Fukue volcano provide information about the variations in the D/H ratios of fluids released from the cold subducting Pacific slab at depths from ~ 90 to ~ 550 km. Notably, the slab depth of ~ 300 km for Rishiri is one of the deepest among arc volcanoes globally, and Fukue is one of the rare active volcanoes that originated from the magmatism rooted in the MTZ.

Numerous scoria samples were collected from various tephra fall deposits at different eruption stages for each of the six volcanoes, and the deposits containing scoria with the highest-quality olivine-hosted melt inclusions were carefully selected. The melt inclusion-bearing olivine crystals were then separated from the scoria samples collected from the selected deposits. To carefully examine the effect of the post-entrapment modification of melt inclusions, volatile content analyses were conducted on at least 25 melt inclusions for each volcano (37 for Iwate, 30 for Akita-Komagatake, 25 for Me-Akan, 38 for Oshima-Oshima, 48 for Rishiri, and 43 for Fukue). Hydrogen isotopic analyses were conducted primarily on melt inclusions with higher H_2_O contents for each volcano. Some melt inclusions with lower H_2_O contents were also measured to assess the variations in the D/H ratio with varied H_2_O content.

## Results and discussion

The descriptions of the scoria samples used in this study are provided in Supplementary Methods. The samples exhibit basaltic compositions (Supplementary Table S1) and contain olivine as the primary mafic phenocryst phase. The samples from the Iwate, Akita-Komagatake, Me-Akan, Oshima-Oshima, and Rishiri volcanoes show trace element concentration patterns that are characteristic of arc basalt. In contrast, the trace element concentration pattern for the samples from the Fukue volcano has an affinity to intra-plate basalt (Supplementary Fig. S1). The Pb isotopic compositions of all the samples lie within the triangular field defined by three subduction zone components: sediment, altered oceanic crust, and depleted mantle (Supplementary Fig. S2).

Volatile (H_2_O, CO_2_, F, S, and Cl) and P_2_O_5_ contents and hydrogen isotopic compositions were analysed on melt inclusions using the ion microprobe (Cameca IMS-1280HR, Ametek Cameca) at the Kochi Institute for Core Sample Research, JAMSTEC. As most measured melt inclusions were glassy without daughter minerals (Supplementary Fig. S3), we did not homogenise the inclusions at high temperatures. The details of the analytical procedures, including sample preparation, analytical conditions, and standard data, are provided in Refs.^20,21^ and “Methods”. The volatile and major elemental compositions and the δD‰ values of the melt inclusions are listed in Supplementary Table S2.

It is well established that the D/H ratios of olivine-hosted melt inclusions do not necessarily represent those of mantle-derived magmas^9,11,16,17^. Mantle-derived magma is not saturated with volatiles at deep levels because volatiles are more soluble in melt at higher pressures. During the ascent of the magma in the crust, the magma becomes saturated with volatiles and vesiculation occurs. Degassing of the magma can cause fractionation of the D/H ratio^22^ because the D/H ratio of an H_2_O-rich vapor phase is higher than that of the melt phase. In addition, during residence of the magma in a crustal magma chamber, the D/H ratio of the magma may be modified by crustal assimilation. Because of these, the D/H ratios of the melt inclusions entrapped in olivine phenocrysts after magma degassing and/or crustal assimilation can be significantly different from those of the original magma. As the magma continues to further ascend, the H_2_O content of the melt progressively decreases. The D/H ratios of olivine-hosted melt inclusions that were entrapped before the magma degassing can be modified by the re-equilibration of the olivine with the surrounding melt because of faster diffusion of H relative to D through the host olivine^16,17^. For these reasons, to extract the information about the mantle processes from melt inclusion data, it is necessary to carefully evaluate the effects of shallow-level processes on the D/H ratios.

The volatile content data are shown in CO_2_‒H_2_O diagrams for all the melt inclusions from the individual volcanoes in Supplementary Figs. S4‒S9. At all the volcanoes, the melt inclusions have variable H_2_O contents, and those with relatively higher H_2_O contents have highly variable CO_2_ contents at a given H_2_O content. Some melt inclusions have shrinkage bubbles, and, therefore, the original CO_2_ contents would have been even higher^17^. CO_2_ is the first compound to be lost by degassing during magma ascent from the upper mantle because it is the least soluble species in silicate melt among the major volatile elements^23^. Therefore, the marked variation in CO_2_ content at a given H_2_O content suggests that the melt with high CO_2_ and high H_2_O contents experienced minimal loss of H_2_O by degassing before the entrapment^24^. In contrast, the low CO_2_ and low H_2_O contents of some melt inclusions may be explained by the entrapment of melts that were degassed during magma ascent (i.e. open-system and closed-system degassing trends in Supplementary Fig. S6a). The high CO_2_ melt inclusions trapped at high pressures might have experienced re-equilibration with lower H_2_O melt through the host olivine at lower pressures, as explained above. δD‰ values of the melt inclusions are expected to correlate negatively with the H_2_O contents when a diffusive loss of H_2_O occurs significantly from the melt inclusions^16,17^. At all the volcanoes, some melt inclusions with lower H_2_O contents tend to have higher δD‰ values, and they might have been affected by diffusive re-equilibration. However, the δD‰ values and the H_2_O content of CO_2_-rich melt inclusions do not exhibit a significant negative correlation, suggesting that CO_2_-rich melt inclusions did not experience significant diffusive re-equilibration. This is consistent with the observation that the δD‰ values of the high CO_2_ melt inclusions are essentially constant irrespective of the CO_2_ contents (Supplementary Figs. S4‒S9). Based on these evaluations, the data of the melt inclusions with the three highest CO_2_ contents were selected as the representative data sets for each volcano.

Of the remaining melt inclusions, some inclusions with high H_2_O and lower CO_2_ contents may also preserve the original D/H ratios, because they experienced minimal loss of H_2_O by degassing from the original magma with high CO_2_ and high H_2_O contents (see the degassing trends in Supplementary Fig. S6a). However, these inclusions were entrapped at lower pressures than those with high CO_2_ contents, and there would be a possibility that the magmas experienced additional processes such as crustal assimilation before the entrapment at the shallow levels. Therefore, the data of the inclusions with low CO_2_ contents were not used as the representative data sets for each volcano.

**Figure 2:**
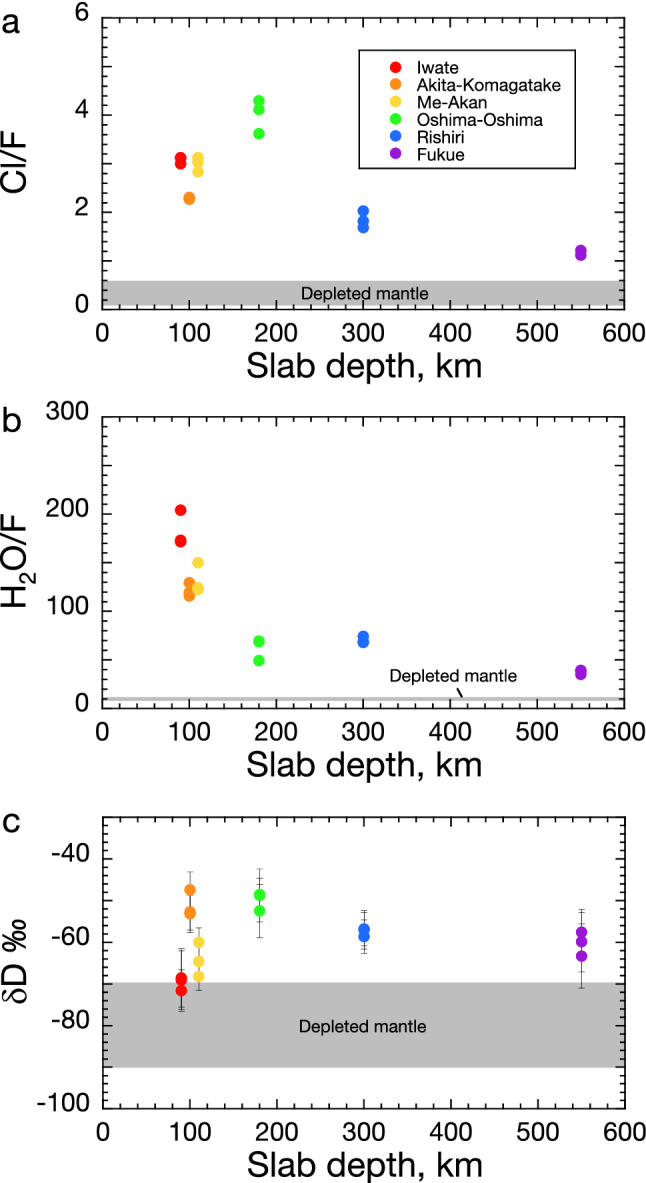
(**a**) Cl/F ratios, (**b**) H_2_O/F ratios, and (**c**) δD‰ values of the representative olivine-hosted melt inclusions from the Iwate, Akita-Komagatake, Me-Akan, Oshima-Oshima, Rishiri, and Fukue volcanoes, plotted as a function of the depth of the subducting Pacific Plate. The values of the depleted mantle (or the majority of MORB) for the Cl/F ratios, H_2_O/F ratios, and δD were sourced from Ref.^25^, Ref.^26^, and Ref.^7^, respectively.

The average H_2_O contents of the three representative inclusions are 3.5 wt.% for Iwate, 2.7 wt.% for Akita-Komagatake, 3.8 wt.% for Me-Akan, 4.1 wt.% for Oshima-Oshima, 3.8 wt.% for Rishiri, and 2.0 wt.% for Fukue. The Cl/F and H_2_O/F ratios of the melt inclusions are shown as a function of the slab depth in Fig. 2a,b, respectively. These ratios are significantly higher for all the volcanoes than those of the depleted mantle or the majority of mid-ocean ridge basalts (MORB)^25,26^. This observation reconfirms that the fluids derived from the Pacific slab were involved in the magma genesis for the volcanoes. In particular, the involvement of the slab-derived fluids at the Fukue volcano supports the ‘Big mantle wedge model’^27,28^, according to which deep dehydration from the stagnant slab in the MTZ plays a primary role in the overlying volcanism. Notably, the slab fluids for the Fukue volcano were not derived from the Philippine Sea Plate because the plate is too hot to efficiently carry water to the depths of the MTZ^19^. The Cl/F and H_2_O/F ratios at Fukue (*Z* = ~ 550 km) are lower than those of the frontal-arc Iwate and Me-Akan volcanoes. This can be attributed to preferential partitioning of Cl and H_2_O into slab fluids relative to F over the slab source materials^29^.

The δD‰ values of the representative melt inclusions are plotted against the slab depths in Fig. 2c. The value of − 80 ± 10‰^7^ was commonly used as the reference δD‰ value for the depleted mantle, and it was later revised to − 60 ± 5‰^30^. However, recent work suggests that the δD‰ values of the depleted mantle could be heterogeneous worldwide, and this value for some depleted MORB, such as those from the North Atlantic, is − 90 ± 10‰^12^. The present study used the traditional δD‰ value of − 80 ± 10‰ for the Pacific depleted mantle. The δD‰ values of the samples from all the volcanoes are higher than those of the depleted mantle, which can be interpreted to result from the addition of D-enriched slab fluids to the source depleted mantle^9^. The water dissolved in a subduction-related primary magma is derived from the H_2_O in the slab-derived fluid and the H_2_O contained in the depleted mantle peridotite. We estimated that the contributions of the slab fluids to the H_2_O budget in the primary magmas from the studied volcanoes range from 92 to 98% (Supplementary Methods), and hence, the δD‰ values of the representative melt inclusions (Fig. 2c) can be regarded as those of the slab-derived fluids at the individual volcanoes.

**Figure 3:**
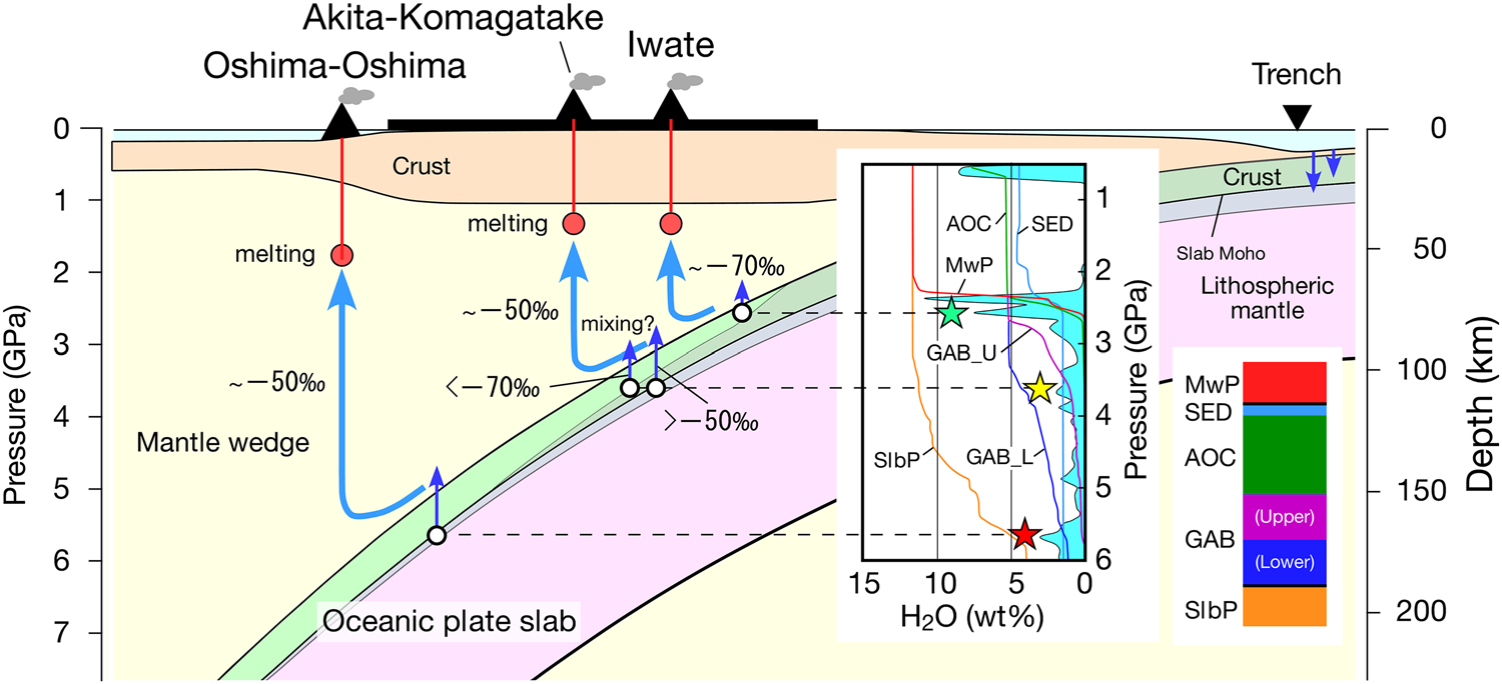
Schematic of the subduction-zone processes in the NE Japan arc. The figure is modified from Fig. 11A of Ref.^42^ illustrating the chemical geodynamics in a cold subduction system. The inset, modified from Fig. 2a of Ref.^31^, shows the result of calculations for the NE Japan arc using the Arc Basalt Simulator version 5. Namely, the results show variations in the total amount of H_2_O released from the slab (the cyan area) and in the amounts of H_2_O stored in the individual layers of the subducting slab with depth. MwP denotes the mantle wedge base peridotite layer just above the subducting slab; SED and AOC refer to the sediment layer and the altered oceanic crust layer, respectively; GAB_U and GAB_L denote the upper and lower gabbro layers, respectively; and SlbP refers to the slab peridotite layer. The green, yellow, and red stars indicate the possible main H_2_O sources of the slab fluids for Iwate, Akita-Komagatake, and Oshima-Oshima, respectively. The red circles denote the melt segregation depths obtained by Refs.^43,44^ (the segregation depth at Oshima-Oshima is assumed to be similar to that at Sannome-gata^43^). Fluids released from the slab surface may be dragged to a slightly deeper depth because they can be trapped in a triple junction of the olivine grains in the overlying mantle wedge base peridotite layer (MwP)^31^. This figure was created using Canvas 14.

One of the crucial observations is that the δD‰ values of Iwate are lower than those of Akita-Komagatake, which is located just behind the frontal arc of the Iwate volcano. This observation cannot be explained by the single reservoir model proposed in Ref.^9^. When the subducting slab behaves as a single H_2_O reservoir, the D/H ratios of the slab fluids would decrease with increasing slab depth because of the continuous release of D-enriched fluids from the slab. Therefore, our observation requires the involvement of at least two H_2_O reservoirs with different D/H ratios in the generation of slab fluids beneath the Iwate and nearby Akita-Komagatake volcanoes. Recently, the role of the slab peridotite layer as the H_2_O source for subduction zone magmas has been receiving increased attention^11,12,31^. The crust portion of the subducting slab, consisting of altered basaltic rocks [altered oceanic crust (AOC) and gabbro (GAB)] and sediment (SED), and the overlying mantle wedge base peridotite (MwP) are considerably dehydrated before reaching the depth beneath the volcanic front (Fig. 3). Contrastingly, the underlying peridotite portion of the slab (SlbP), which is effectively hydrated during bending at the trench, can survive as a water reservoir beneath the arc^12,31^ (Fig. 3). This scenario can explain the observed spatial variation in the δD‰ data. When the slab fluid for Iwate was supplied primarily from the crust portion of the slab (green star in Fig. 3), the D/H was likely low because this portion had already been considerably dehydrated at shallower levels. Conversely, the main source of the fluid beneath the Akita-Komagatake volcano is considered to have been the underlying peridotite portion of the slab (yellow star in Fig. 3). In this case, the fluid would be relatively D-enriched because this portion was not significantly dehydrated before reaching ~ 100 km depth. Therefore, considering that the initial D/H ratios of the crust and peridotite portions of the slab were similar at the time of the subduction, the fluid released at ~ 2.5 GPa from the crust portion would have lower D/H ratios than that released at ~ 3.5 GPa from the underlying peridotite portion^11,12^. The low δD‰ values of the Me-Akan samples (− 60 to − 70‰) may also reflect that the slab fluid responsible for the magmatism was sourced primarily from the crust portion of the slab, considering that the volcano is located at the volcanic front of the Kuril arc.

The δD‰ values of the melt inclusions from the Mariana arc basalts are high, ranging from − 55 to − 12‰^9^, compared to those of the frontal-arc Iwate and Me-Akan volcanoes. This can be attributed to the fact that the low-δD fluid from the dehydrated crustal portion of the slab did not significantly contribute to the Mariana arc magmatism because of the deeper slab depths (~ 115 km)^32^ of the Mariana arc volcanoes. Alternatively, it is possible that the contribution of the low-δD fluid was obscured by the main high-δD fluid flux from the less-dehydrated peridotite portion of the slab because of the high dip angle (70°‒80°) of the Pacific slab in the Mariana arc compared with shallower angles (30°‒40°) in the NE Japan and Kuril arcs^33^.

The δD‰ values of Rishiri (*Z* = ~ 300 km) are similar to those of Fukue (*Z* = ~ 550 km). At levels deeper than ~ 150 km, the peridotite (SlbP) and gabbro (GAB_L) portions of the slab would be the main H_2_O source^31,34^, and water is supplied primarily by the breakdown of serpentine in the peridotite layer^12^. No significant decrease in the δD‰ values of slab fluids deeper than ~ 300 km suggests that significant dehydration of the peridotite portion of the slab did not occur during the subduction to the MTZ. This result is consistent with the observation that only a few instances of magmatism, including those of Rishiri and Fukue, are caused by a fluid release from the Pacific slab with *Z* > ~ 200 km. The host basalts of these samples (i.e. Rishiri and Fukue) have significantly enriched Pb isotopic features compared with the depleted mantle (Supplementary Fig. S2). This suggests that the fluids that were dehydrated from the peridotite portion of the slab interacted with the overlying crust portion (i.e. ‘slab rehydration’^12^) before the release to the wedge mantle.

Because δD‰ values of ~ − 60‰ at Fukue are representative of the slab fluid beneath the Fukue volcano (Supplementary Method), the δD‰ values of the residual slab materials that were stored in the MTZ can be estimated to be ~ − 90‰, assuming the D/H fractionation factor (1000 lnα) between the slab and fluid of − 30‰ (Ref.^9^ and the references therein). If we consider the possible pressure dependence on the fractionation factor^35^, the δD‰ of the residual slab materials could be higher than ~ − 90‰. Our new estimate of the δD‰ values for the recycled slab materials is much higher than − 234‰ estimated by Ref.^9^ and is also slightly higher than − 100‰ to − 120‰ obtained by Ref.^11^. At Koolau volcano (Hawaii) where recycled slab materials are considered to have contributed significantly to the source mantle^36^, a δD‰ value of − 120‰ was estimated^16^. If the δD‰ value of the Earth’s primordial H_2_O is − 60 to − 80‰^13,37^, then the lower δD of − 120‰ compared to both the primordial δD and the δD‰ value of > − 90‰ for slab materials stored in the MTZ may suggest that the recycled materials in the Koolau basalts experienced further D/H fractionation during the transport from the MTZ to the bottom of the mantle as a cold plume and or during the residence at the core-mantle boundary until the ascent as a hot plume.

## Methods

### Analytical methods

Whole-rock major and trace element analyses as well as Sr, Nd, and Pb isotopic analyses on the scoria samples were carried out at the Faculty of Science, Hokkaido University, Japan. Major element compositional analyses of melt inclusions and the host olivine crystals were also conducted at Hokkaido University. The details of the analytical methods were provided in Supplementary Methods.

The CO_2_, H_2_O, F, Cl, S, and P_2_O_5_ content analyses of the melt inclusions were conducted using an ion microprobe CAMECA IMS-1280HR, AMETEK CAMECA, at the Kochi Institute for Core Sample Research, JAMSTEC, following the methods described in Ref.^20^. Melt inclusion-bearing olivine crystals separated from the scoria samples were individually mounted in an acrylic resin, and they were polished until melt inclusions were exposed. The polished crystals were then mounted in an indium-filled Al-disc of 1 inch to minimise volatile background. To dry the sample completely, the cleaned Al-disc was kept in an oven at 80 °C and 10^–7^ torr for more than 48 h prior to Au coating. The Au-coated Al-disc was stored in the airlock chamber of the spectrometer at < 10^–8^ torr overnight prior to the analyses.

We used a 20 keV Cs^+^ primary beam of 500 pA defocused to a diameter of 10–15 µm. A − 10 keV electron gun of ~ 100 μm in diameter was used to avoid charging the sample surface. We set the field aperture size to 5 × 5 µm of the field of view of the secondary ion image to eliminate secondary ion signals from surface contamination. The instrument was operated with a mass resolving power of ~ 6000, which allows us to distinguish completely the interferences of ^34^S^1^H on ^35^Cl, ^17^O on ^16^O^1^H, ^29^Si^1^H on ^30^Si, and ^31^P^1^H on ^32^S. We detected negative secondary ions of ^12^C, ^16^OH, ^19^F, ^30^Si, ^31^P, ^32^S, and ^35^Cl by an axial electron multiplier using a magnetic peak switching method. The total measurement time for each analysis, including 20 s for pre-sputtering, 120 s for auto-centring of secondary ions, and 10 cycles for measurements, was ~ 7 min. Fourteen in-house basaltic glass standards with broad ranges of volatile contents were used^20^. The concentrations of volatiles and P_2_O_5_ in melt inclusions were determined by calibration curves of the ^30^Si-normalised intensities of volatile elements and ^31^P of a set of basaltic standards. Analytical reproducibilities of CO_2_, H_2_O, F, P_2_O_5_, S, and Cl concentrations were 3.6, 0.8, 2.0, 0.9, 0.8, and 1.3%, respectively, which were obtained by repeated analyses (n = 72) on different glass fragments (n = 38) of a homogeneous MORB basalt (EPR-G3) over seven analytical sessions. The maximum uncertainties were less than 5% for H_2_O, 15% for CO_2_, and 10% for Cl, F, S, and P_2_O_5_.

The hydrogen isotope analyses were conducted on the same melt inclusions as those used for the volatile content analyses. The same microprobe as above was used, and a method described in Ref.^21^ was followed. We used a 20 keV Cs^+^ ion beam of ~ 5 nA defocused to ~ 15 µm in diameter. A − 10 keV electron gun of ~ 100 µm in diameter was used. We set the field aperture size to 7 × 7 µm, which is smaller than the beam size, to minimise hydrogen contamination from the edge of the primary beam. We measured negative secondary ions of ^16^OH^−^ and ^16^OD^−^ in multi-detection mode with a Faraday cup (FC) of 10^+11^ or 10^+12^ Ω resistance and axial electron multiplier, respectively. To sufficiently separate the ^16^OD^−^ from ^18^O^−^, ^17^OH^−^, and ^16^OHH^−^, we set the mass resolving power (M/∆M) of the axial detector to approximately 10,000. The mass resolving power of the FC detector was set to ~ 5000 with a slit of 250 µm in width, allowing sufficient separation of the ^16^OH^−^ signal from the ^17^O^−^ signal. The total measurement time for each analysis, including 20 s pre-sputtering, ~ 120 s for auto-centring of ^16^OH^−^ to the field and contrast apertures, and 4 s × 50 cycles for measurements, was ~ 6 min. FC background stability was ~ 700 cps (2sd), which did not affect the D/H ratios as long as OH was > 0.4 Mcps or H_2_O was > 0.2 wt.%.

Three in-house basaltic glass standards were used for the calibration of the analyses. The water contents and δD‰ values are 0.54 wt.% and − 107 ± 20‰ (2sd), 0.94 wt.% and − 109 ± 3‰, and 3.6 wt.% and 37 ± 5‰, respectively. The δD‰ values were determined by TCEA/IRMS (high-temperature conversion elemental analyser/isotope ratio mass spectrometer). The standard bracketing method was adopted to determine the D/H ratios of the melt inclusions. The working standard of Hawaiian submarine basaltic glass (H_2_O content of 1.4 wt.% and δD‰ of − 95 ± 5‰) was embedded in the sample mount, and it was measured five times for every ten unknown samples. We calculated the instrumental mass fractionation factor and analytical error by averaging 10 data points of the standard glass. If the 2se of the melt inclusion was greater than the 2sd, we adopted the former as the analytical error; otherwise, the latter was adopted as the analytical error. Water contents of the melt inclusions were typically higher than that of the working standard in this study, and hence, analytical errors of most melt inclusions were 2sd of the working standard averages of each bracket.

## Supplementary Information

Supplementary Information.

Supplementary Tables.

## Data Availability

The datasets generated and/or analysed during the current study are available from the corresponding author upon reasonable request.
