# Supplementary material for: Tracing the subducting Pacific slab to the mantle transition zone with hydrogen isotopes
Source: Sci Rep. 2021 Sep 21;11:18755. doi: 10.1038/s41598-021-98307-y (PMC8455532; doi:10.1038/s41598-021-98307-y)
Supplement: Supplementary file 1 — Supplementary Information. [file 41598_2021_98307_MOESM1_ESM.pdf]

Supplementary Information

Article in *Scientific Reports*

Tracing the subducting Pacific plate to the mantle transition zone with hydrogen isotopes

Takeshi Kuritani[1]*, Kenji Shimizu[2], Takayuki Ushikubo[2], Qun-Ke Xia[3], Jia Liu[3], Mitsuhiro Nakagawa[1], Hajime Taniuchi[4], Eiichi Sato[5], Nobuo Doi[6]

[1]Department of Earth and Planetary Sciences, Faculty of Science, Hokkaido University, Sapporo, Japan

[2]Kochi Institute for Core Sample Research, Japan Agency for Marine-Earth Science and Technology, Nankoku, Japan

[3]Key Laboratory of Geoscience Big Data and Deep Resource of Zhejiang Province, School of Earth Sciences, Zhejiang University, Hangzhou, China

[4]Department of Natural History Sciences, Hokkaido University, Sapporo, Japan

[5]Earth Science Laboratory, Hokkaido University of Education, Asahikawa, Japan

[6]Research Center for Regional Disaster Management, Iwate University, Morioka, Japan

*Corresponding author: kuritani@sci.hokudai.ac.jp

Supplementary Figures

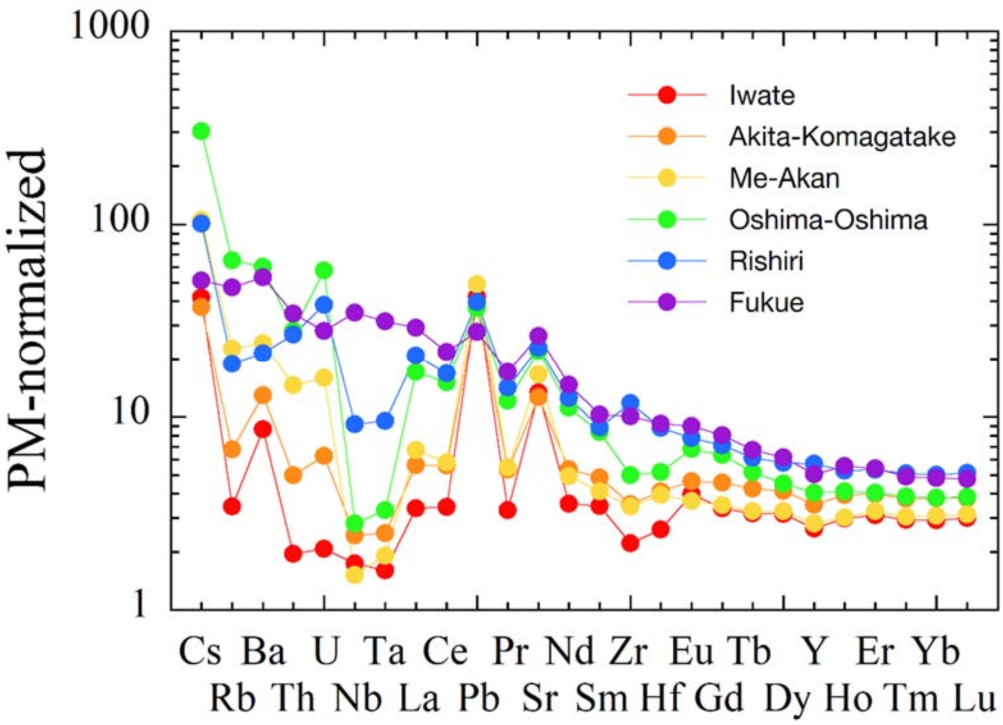

Supplementary Figure S1. Primitive mantle (PM)-normalised multi-element concentration diagram for the scoria samples from the six studied volcanoes. The trace element concentrations of the primitive mantle were sourced from ref.[1].

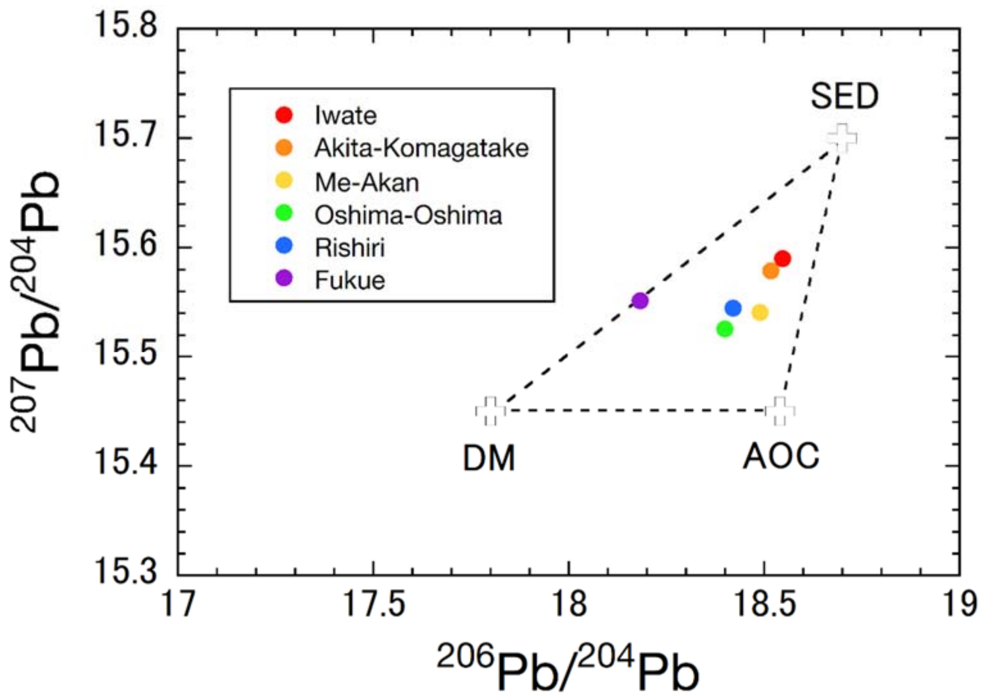

Supplementary Figure S2. Pb isotopic compositions of the scoria samples from the six studied volcanoes. The compositions of the geochemical components, including depleted mantle (DM), altered oceanic crust (AOC), and sediment (SED), were sourced from ref.[2].

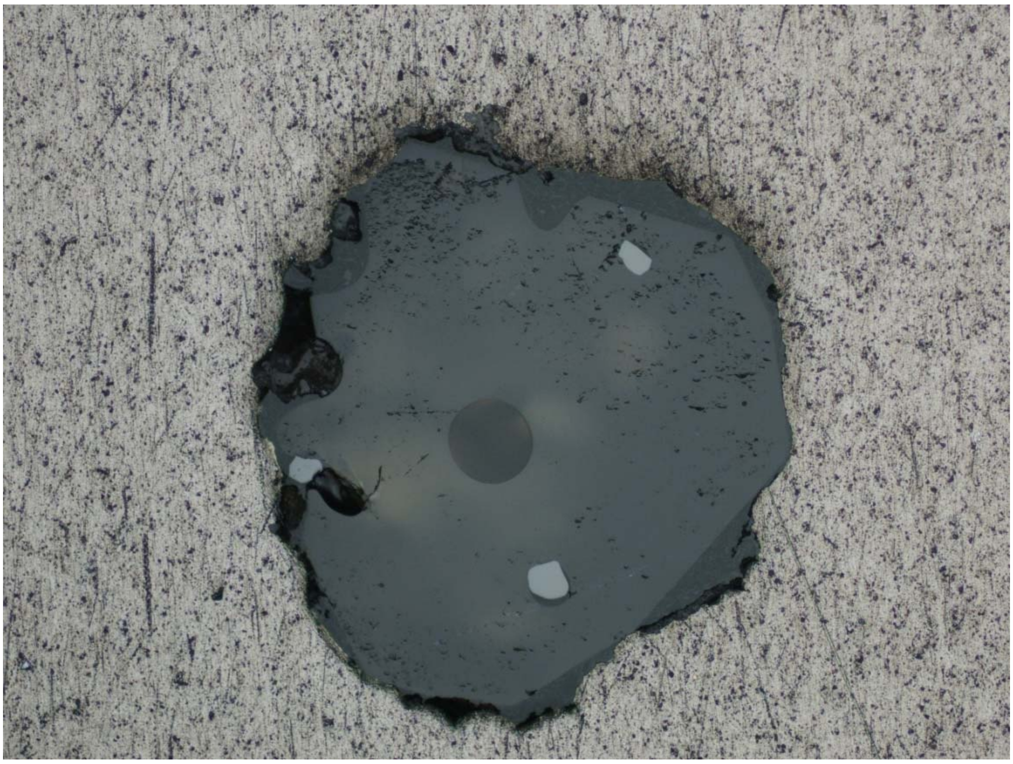

Supplementary Figure S3. Photomicrograph of an olivine phenocryst with a melt inclusion from sample MI5_#22, Iwate volcano (reflected light), mounted on an indium holder. The inclusion is glassy and free from quenched crystals. The diameter of the melt inclusion is ~100 μm.

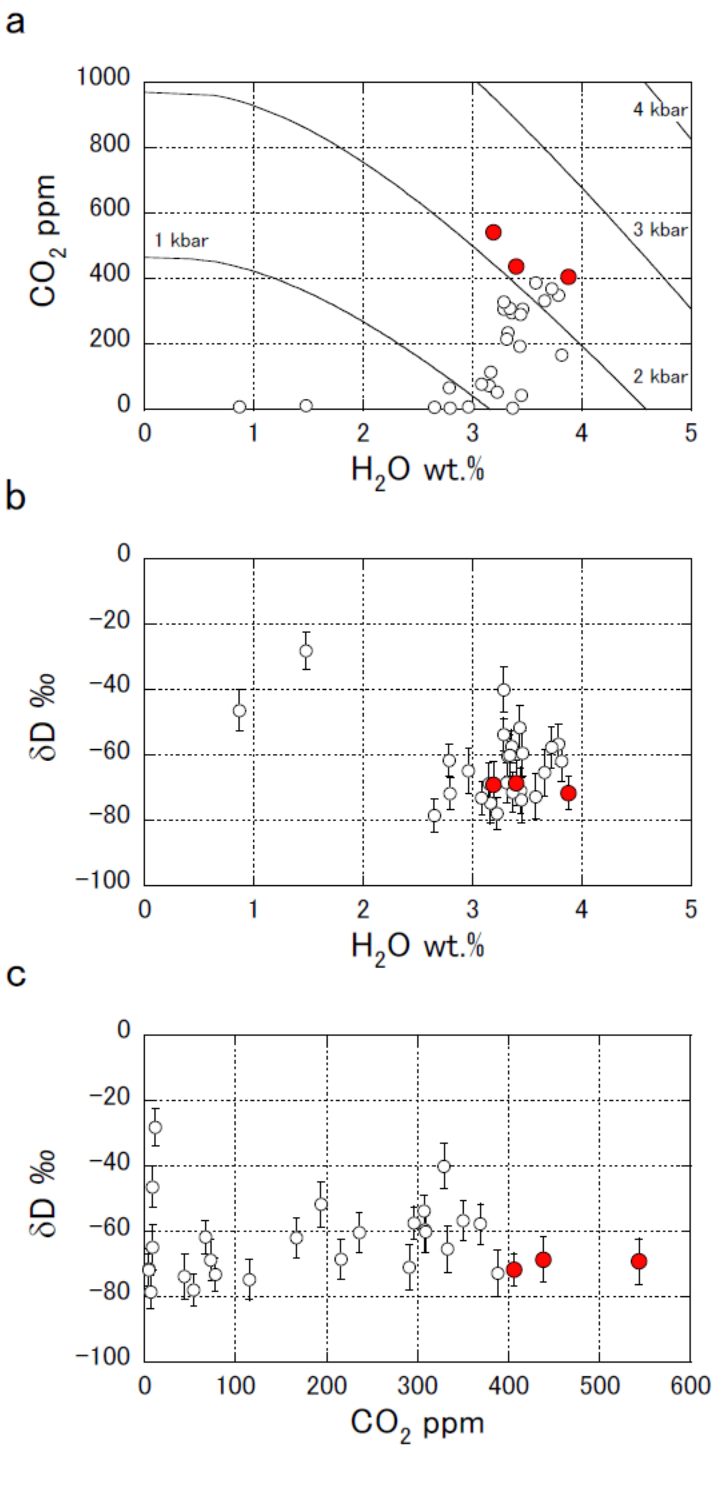

Supplementary Figure S4. Compositions of olivine-hosted melt inclusions from the Iwate volcano shown in (a) CO_2–H_2O, (b) δD–H_2O, and (c) δD–CO_2 diagrams. The isopleths in (a) were drawn using the model given by ref.[3]. The red plots indicate the data of the three highest CO_2 contents.

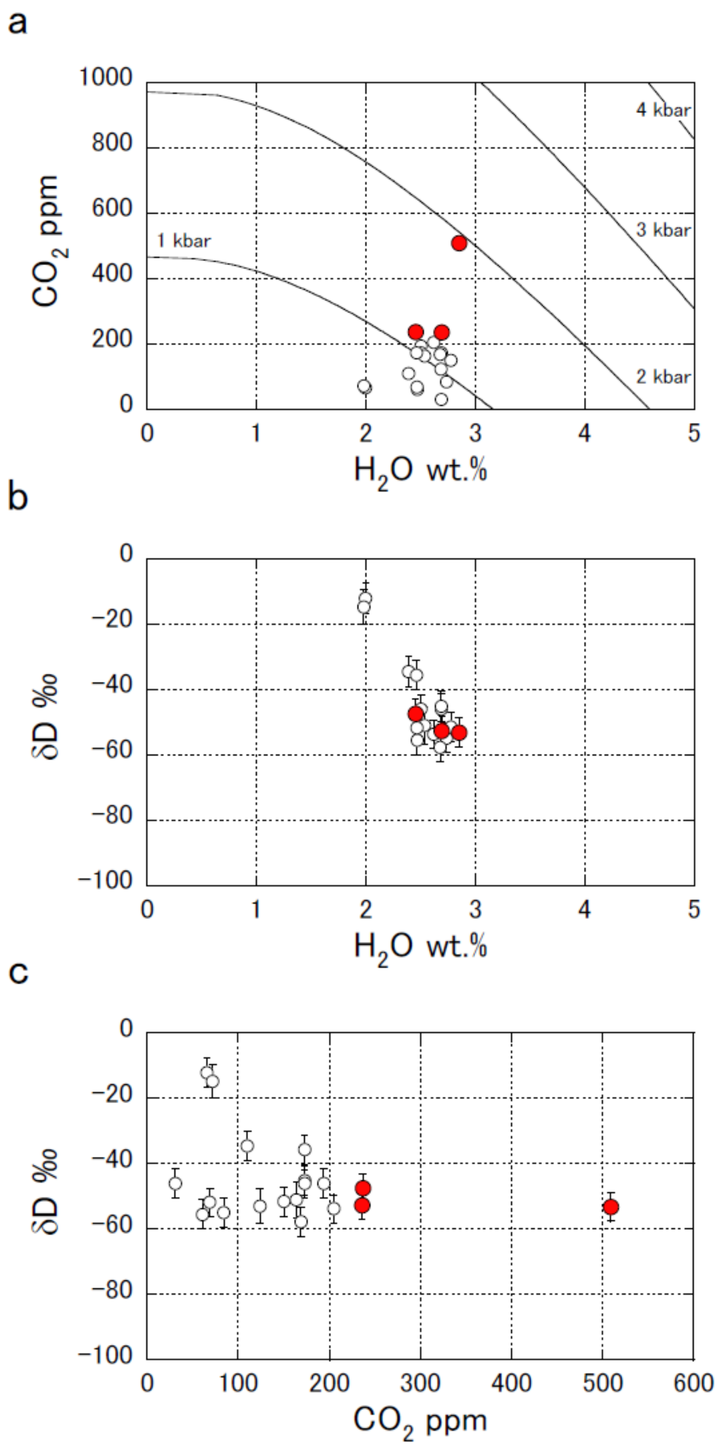

Supplementary Figure S5. Compositions of olivine-hosted melt inclusions from the Akita-Komagatake volcano shown in (a) CO_2–H_2O, (b) δD–H_2O, and (c) δD–CO_2 diagrams. The isopleths in (a) were drawn using the model given by ref.[3]. The red plots indicate the data of the three highest CO_2 contents.

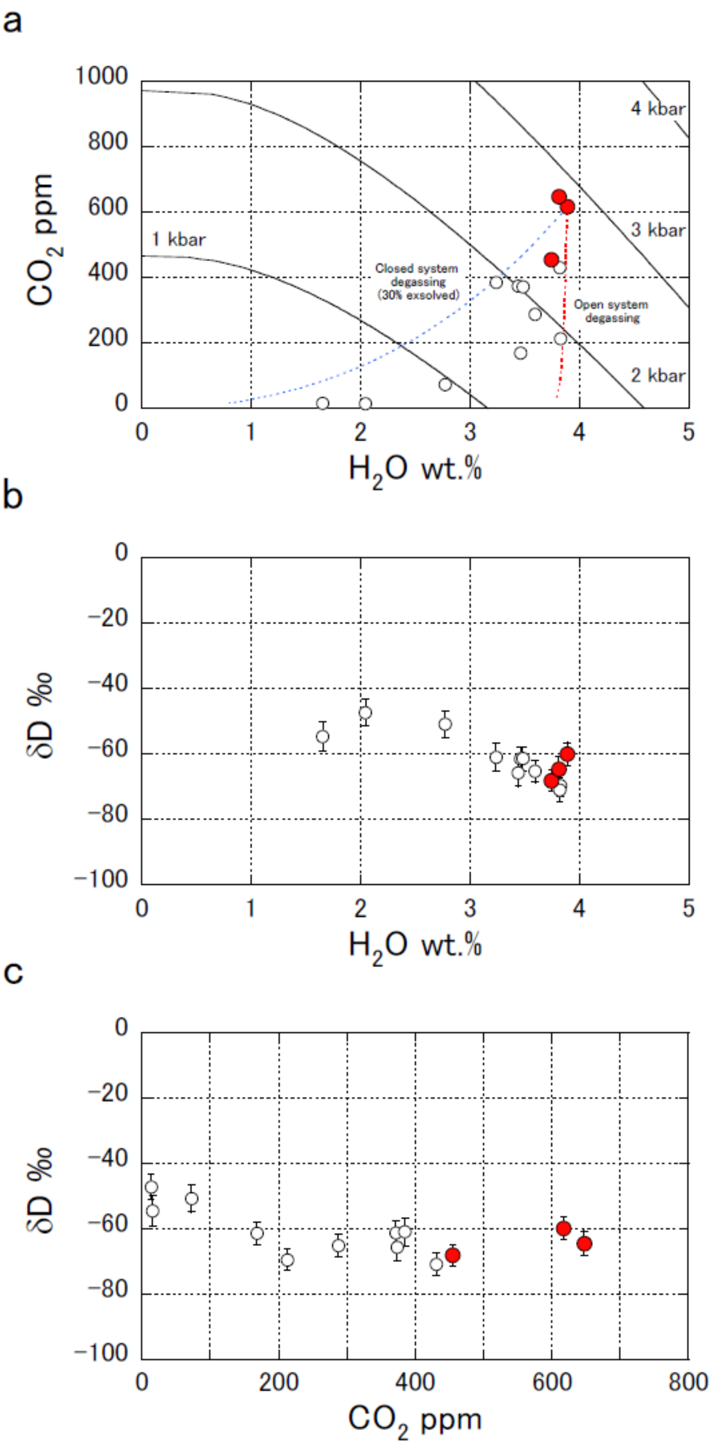

Supplementary Figure S6. Compositions of olivine-hosted melt inclusions from the Me-Akan volcano shown in (a) CO_2–H_2O, (b) δD–H_2O, and (c) δD–CO_2 diagrams. The isopleths and degassing trends in (a) were drawn using the model given by ref.[3]. The red plots indicate the data of the three highest CO_2 contents.

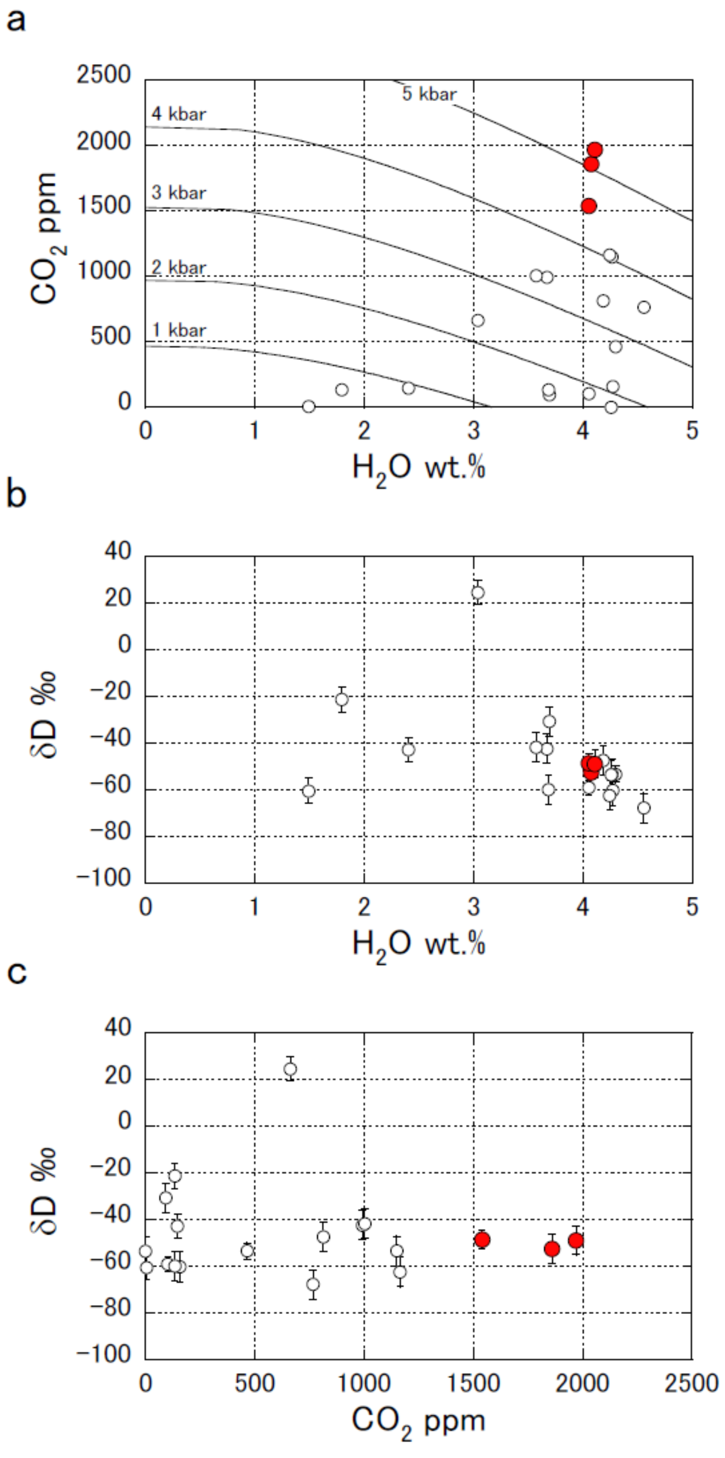

Supplementary Figure S7. Compositions of olivine-hosted melt inclusions from the Oshima-Oshima volcano shown in (a) CO_2–H_2O, (b) δD–H_2O, and (c) δD–CO_2 diagrams. The isopleths in (a) were drawn using the model given by ref.[3]. The red plots indicate the data of the three highest CO_2 contents.

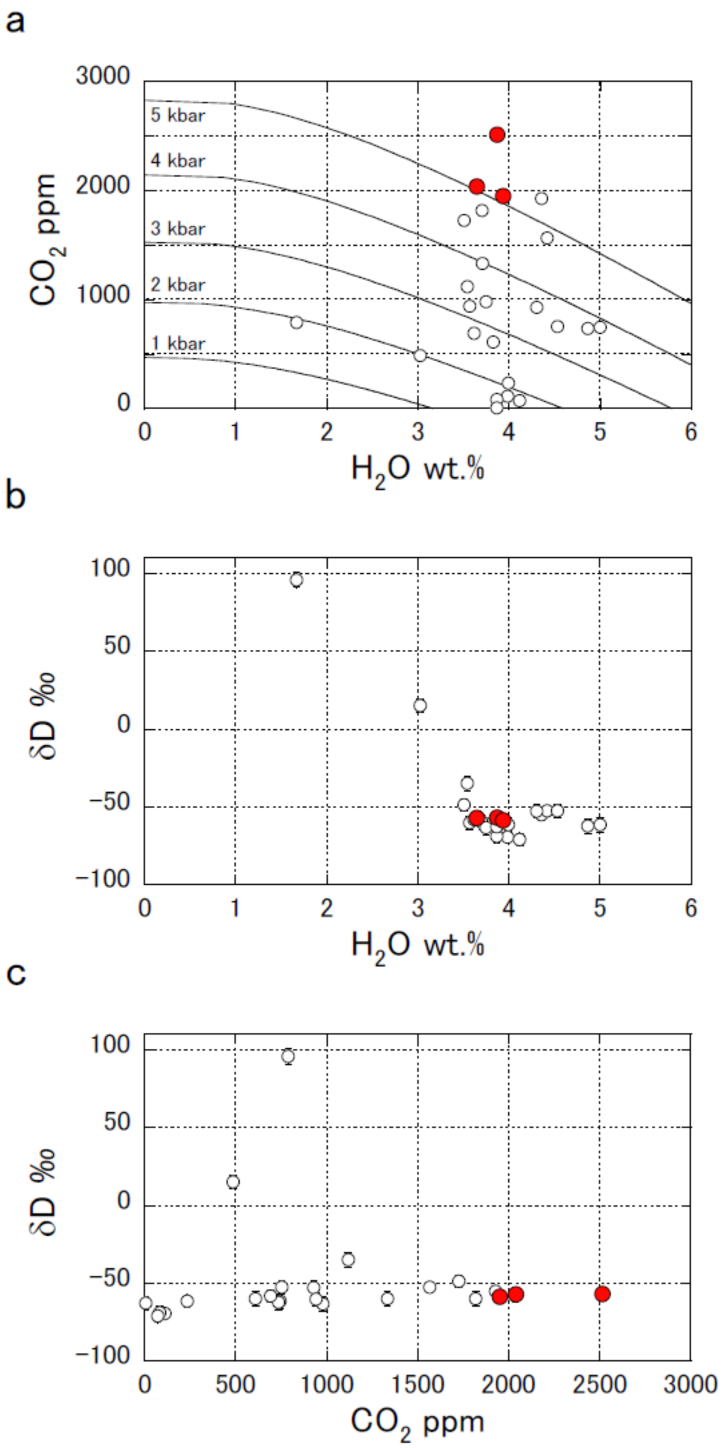

Supplementary Figure S8. Compositions of olivine-hosted melt inclusions from the Rishiri volcano shown in (a) CO_2–H_2O, (b) δD–H_2O, and (c) δD–CO_2 diagrams. The isopleths in (a) were drawn using the model given by ref.[3]. The red plots indicate the data of the three highest CO_2 contents.

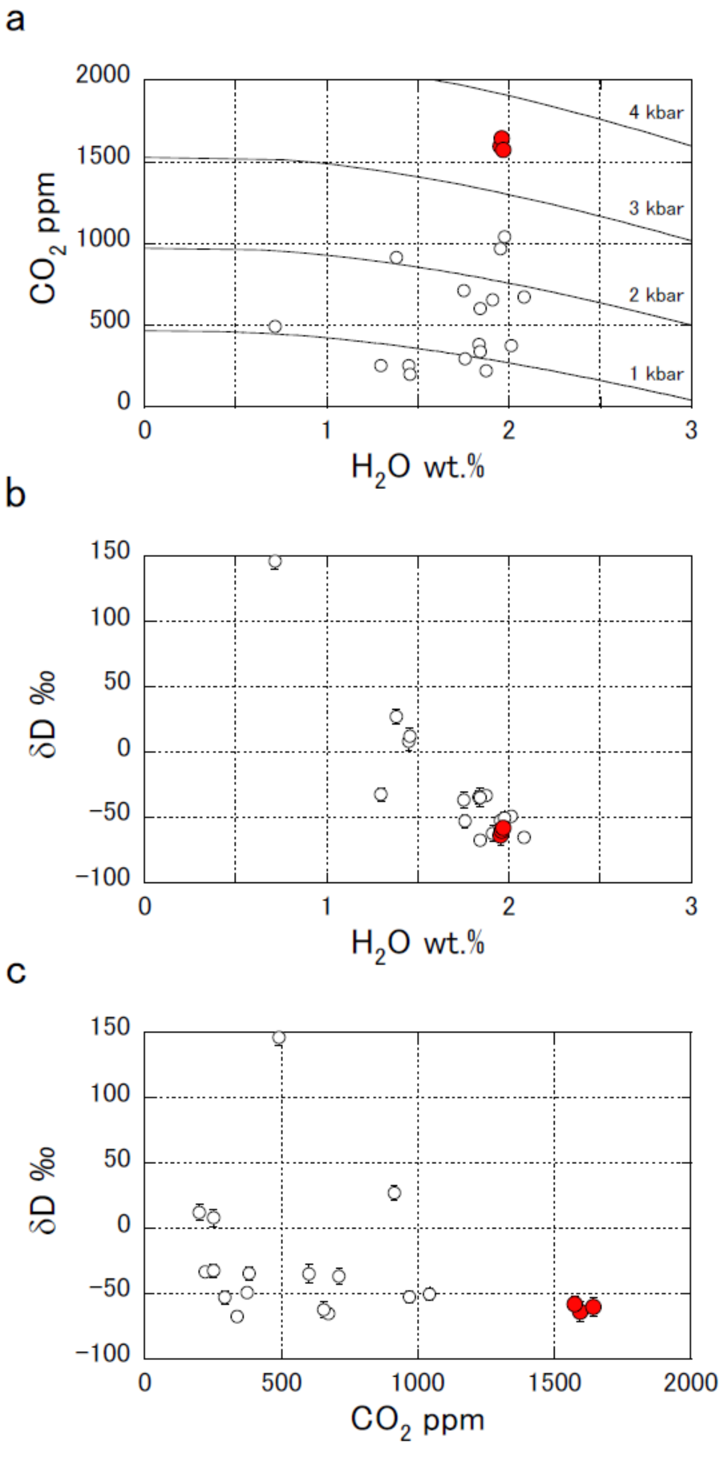

Supplementary Figure S9. Compositions of olivine-hosted melt inclusions from the Fukue volcano shown in (a) CO_2–H_2O, (b) δD–H_2O, and (c) δD–CO_2 diagrams. The isopleths in (a) were drawn using the model given by ref.[3]. The red plots indicate the data of the three highest CO_2 contents.

Supplementary Methods

Sample descriptions

Iwate is an active polygenetic volcano located on the volcanic front of the NE Japan arc. Quaternary basalt and andesite comprise a large part of the volcano[4]. The sample was collected from the scoria fall deposits (Kariya scoria) derived from the summit eruption in AD 1686. Volatile contents on olivine-hosted melt inclusions from the scoria of this stage were reported by ref.[5]. The sample contains ~25% plagioclase phenocryst, ~5% olivine phenocrysts, and rare pyroxene phenocrysts.

Akita-Komagatake is an active polygenetic volcano located ~30 km from the volcanic front in the NE Japan arc. The volcanic edifice is primarily composed of Quaternary basalt and andesite[6]. The sample was collected from the scoria fall deposits of the Hokubu 2nd pyroclastic cone (~7 ka[6]), and contains ~15% plagioclase phenocryst, ~5% olivine phenocrysts, and rare pyroxene phenocrysts.

Me-Akan is an active polygenetic volcano located on the volcanic front of the southern Kuril arc. The volcano has been active since the Late Pleistocene[7]. The sample was collected from the scoria fall deposits ejected from a stratovolcano called Akan-Fuji located on the southern part of the Me-Akan volcano. The formation age of Akan-Fuji is 1–2 ka[8]. The sample contains ~10% plagioclase phenocryst, ~3% olivine phenocrysts, and rare clinopyroxene phenocrysts.

Oshima-Oshima is an active volcano located at the rear of the NE Japan arc. The volcano came into existence in the Late Pleistocene, and the recent eruption occurred in AD 1759 (ref.[9]). The sample was collected from the scoria fall deposits on the volcano, and the eruption age is AD 1741. The sample contains ~10% olivine phenocrysts, ~5% plagioclase phenocrysts, and ~3% clinopyroxene phenocrysts.

Rishiri is an active volcano located at the rear of the Kurile arc. The volcano has been active since ~0.2 Ma[10]. The sample was collected from a cinder cone called Araragiyama situated on the southern flank of the main stratovolcano. This cinder cone originated at the vent of the Araragiyama lava flows[10]. Although the eruption age has not been determined, the products are considered to be younger than 20 ka[11]. The sample contains ~5% olivine phenocrysts and rare plagioclase phenocrysts.

The Fukue Volcanic Group, which consists of many monogenetic volcanoes, is located in the southwestern part of Japan. The volcanic activity began at ~0.5 Ma, and the recent activity is estimated to have occurred at 2.3–2.4 ka[12]. The sample was collected from the scoria fall deposits on the Akashima volcano[13]. Although the age of this volcano has not been determined, it is among the youngest volcanoes in the Fukue

Volcanic Group, because the eruptive products are fresh[14]. The sample contains ~5% olivine phenocrysts with minor (<0.5%) plagioclase phenocrysts.

Analytical methods

Whole-rock major and trace elemental analyses as well as Sr, Nd, and Pb isotopic analyses on the scoria samples were carried out at the Faculty of Science, Hokkaido University, Japan. The rock specimens were crushed to coarse chips with diameters of 3–5 mm. The chips were rinsed with deionised water in an ultrasonic bath for >5 h and then dried at 110°C for >12 h. The washed chips were powdered with an alumina rod in a polycarbonate vessel using a Yasui Kikai multi-beads shocker. The concentrations of the whole-rock major elements and some trace elements (Sc, V, Cr, Co, and Ni) were determined by X-ray fluorescence using a Spectris MagiX PRO (Panalytical Ltd.). The powdered samples were ignited at 900°C for >12 h in a muffle furnace, and loss on ignition was determined gravimetrically. Glass beads were prepared by fusion with an alkali flux (sample dilution: 2:1) consisting of a 4:1 mixture of lithium tetraborate and lithium metaborate. The composition of the Geological Survey of Japan reference material JB-3 was measured during this study; the measured and reference values are listed in Supplementary Table S3. The standard deviation of the replicate analyses of JB-3 is also provided in Supplementary Table S3. Additional trace elements were analysed by inductively coupled plasma mass spectrometry (ICP–MS) using a Thermo Fisher Scientific X-series instrument, following the methods of ref.[15]. Replicate analyses of the standard samples established an analytical reproducibility of better than 2% for all the elements[15]. The measured trace elemental concentrations for JB-3 during this study and its reference values are listed in Supplementary Table S3.

We followed previous studies with regard to the analytical procedures for the chemical separation of Sr[16,17], Nd[18], and Pb[19]. The isotopic ratios were determined using multiple collector–ICP–MS (Neptune Plus, Thermo Fisher Scientific). The mass fractionation factors for Sr and Nd were internally corrected using $^{86}Sr/^{88}Sr = 0.1194$ and $^{146}Nd/^{144}Nd = 0.7219$, respectively, and those for Pb were corrected using Tl as an external standard. Additional corrections were performed by applying a standard bracketing method using NIST987, JNdi-1, and NIST981 for the isotopic analyses of Sr, Nd, and Pb, respectively, and normalising to $^{87}Sr/^{86}Sr = 0.710240$ for NIST987, $^{143}Nd/^{144}Nd = 0.512117$ for JNdi-1, and $^{206}Pb/^{204}Pb = 16.9424$, $^{207}Pb/^{204}Pb = 15.5003$, and $^{208}Pb/^{204}Pb = 36.7266$ for NIST981[20]. The isotopic ratios of JB-3 measured during this study, reference values, and standard deviations of the replicate analyses are provided in Supplementary Table S3.

Major elemental compositions of melt inclusions and the host olivine crystals were determined using a JEOL JXA-8800 electron microprobe at Hokkaido University. An accelerating voltage of 15 kV, a beam current of 20 nA, peak and background counting times of 20 s and 10 s, respectively, and a focused beam were adopted for olivine. The following operating conditions were used for glass: an accelerating voltage of 15 kV, a beam current of 10 nA, peak and background counting times of 10 s and 5 s, respectively, and a beam diameter of 30–50 μm. Both oxide and natural mineral standards were used, and the data were obtained using the ZAF correction method.

H_2O budgets in the primary magmas

For interpreting the δD values of the melt inclusions obtained in this study, the relative contributions of slab fluids to the H_2O budget in the primary magmas were approximately estimated at each volcano. It was assumed that the melts with the compositions of the highest CO_2 samples MI5_#29b, MI6_#10, MI4_#31a, MI5_#41, MI6_#41, and MI2_#5a in Iwate, Akita-Komagatake, Me-Akan, Oshima-Oshima, Rishiri, and Fukue, respectively (Supplementary Table S2), were derived from the primary magmas solely by olivine fractionation. The amounts of the olivine crystals that were fractionated from the primary magmas (X_{ol}, wt.%) were then estimated by assuming the $Fe^{3+}/\Sigma Fe$ ratio of the melt of 0.25 (ref.[21]), the equilibrium $(Fe^{2+}/Mg)^{olivine/melt}$ distribution coefficient of 0.3 (ref.[22]), and the Fo content of the mantle olivine of 90. Using the average H_2O contents of the representative melt inclusions (C_{H2O}^{melt}, wt.%) and the amounts of the fractionated olivines (X_{ol}), the H_2O contents of the primary magmas (C_{H2O}^{pri}, wt.%) were obtained (Supplementary Table S4).

At the Iwate, Akita-Komagatake, Rishiri, and Fukue volcanoes, the information about the degrees of mantle melting (F, wt.%) is available in the literatures[11,13,23,24], and they are listed in Supplementary Table S4. For the Me-Akan and Oshima-Oshima volcanoes, the degrees of melting were assumed to be similar to those of the frontal-arc Iwate volcano and the rear-arc Sannome-gata volcano[23], respectively. The H_2O content of the partial melt of the mantle peridotite (C_{H2O}^{perid}, wt.%) (without an influx of slab fluids) can be calculated by a batch melting model using a bulk distribution coefficient for H_2O of 0.012 and an H_2O content for the depleted mantle of 116 ppm[25], as well as the degree of melting (F). Finally, the relative contribution of slab fluid in the H_2O budget of the primary magma ($X^{slab\ fluid}$, %) can be calculated as $(100 - C_{H2O}^{perid}) / C_{H2O}^{pri} \times 100$ (Supplementary Table S4).

Supplementary references

1. Sun, S.-S. & McDonough, W. F. Chemical and isotopic systematics of oceanic basalts: implications for mantle composition and processes. In: Saunders, A.D., Norry, M. J. (Eds.), Magmatism in the Ocean Basins. *Geol. Soc. London, Spec. Pub.* **42**, 313–345 (1989).
2. Kimura, J. Modeling chemical geodynamics of subduction zones using the Arc Basalt Simulator version 5. *Geosphere* **13**, 992–1025 (2017).
3. Newman, S. & Lowenstern, J. B. VolatileCalc: a silicate melt–H_2O–CO_2 solution model written in Visual Basic for excel. *Comput. Geosci.* **28**, 597-604, 2002.
4. Itoh, J. & Doi, N. Geological map of Iwate Volcano. Geological Survey of Japan, National Institute of Advanced Industrial Science and Technology, Tsukuba (2005).
5. Rose-Koga, E. F., Koga, K. T., Hamada, M., Hélouis, T., Whitehouse, M. J. & Shimizu, N. Volatile (F and Cl) concentrations in Iwate olivine-hosted melt inclusions indicating low-temperature subduction. *Earth Planets Space* **66**, 81 (2014).
6. Fujinawa, A., Iwasaki, M., Honda, K., Nagao, A., Wachi, T. & Hayashi, S. Eruption history in the post-caldera stage of Akita-Komagatake Volcano, northeastern Japan Arc: correlation between eruptives constituting volcanic edifices and air-fall tephra layers. *Bull. Volcanol. Soc. Jpn.* **49**, 333–354 (2004).
7. Wada, K., Ikegami, H. & Inaba, T. Chemical compositions of the rocks from Me-akan Volcano, eastern Hokkaido, Japan. *Rep. Taisetsuzan Inst. Sci.* **32**, 43–60 (1998).
8. Ikegami, H. & Wada, K. Eruption history of Akanfuji at Me-akan volcano, eastern Hokkaido. *Programme and abstracts the Volcanological Society of Japan 1994*, 126 (1994).
9. Geological Survey of Japan. Volcanoes of Japan. https://gbank.gsj.jp/volcano/index.htm (2013).
10. Ishizuka, Y. Eruptive history of Rishiri Volcano, northern Hokkaido, Japan. *Bull. Volcanol. Soc. Jpn.* **44**, 23–40 (1999).
11. Kuritani, T., Yokoyama, T. & Nakamura, E. Generation of rear-arc magmas induced by influx of slab-derived supercritical liquids: implications from alkali basalt lavas from Rishiri Volcano, Kurile arc. *J. Petrol.* **49**, 1319–1342 (2008).
12. Nagaoka, S. & Furuyaka, K. Eruptive history of the Onidake Volcano Group on Fukue Island, western Japan. *J. Geography* **113**, 349–382 (2004).

13. Kuritani, T., Sakuyama, T., Kamada, N., Yokoyama, T. & Nakagawa, M. Fluid-fluxed melting of mantle versus decompression melting of hydrous mantle plume as the cause of intraplate magmatism over a stagnant slab: implications from Fukue Volcano Group, SW Japan. *Lithos* **282–283**, 98–110 (2017).
14. Matsui, K., Kamada, Y. & Kurasawa, H. Geology of the Tomie district. Quadrangle Series, scale 1:50,000, Geol. Survey Jpn. 34 pp (1977).
15. Yokoyama, T., Nagai, Y., Hinohara, Y. & Mori, T. Investigating the influence of non-spectral matrix effects in the determination of twenty-two trace elements in rock samples by ICP-QMS. *Geostand. Geoanal. Res.* **41**, 221–242 (2017).
16. Pin, C., Briot, D., Bassin, C. & Poitrasson, F. Concomitant separation of strontium and samarium-neodymium for isotopic analysis in silicate samples, based on specific extraction chromatography. *Anal. Chim. Acta* **298**, 209–217 (1994).
17. Noguchi, T., Shinjo, R., Ito, M., Takada, J. & Oomori, T. Barite geochemistry from hydrothermal chimneys of the Okinawa Trough: insight into chimney formation and fluid/sediment interaction. *J. Mineral. Petrol. Sci.* **106**, 26–35 (2011).
18. Pin, C. & Zalduegui, J. F. S. Sequential separation of light rare-earth elements, thorium and uranium by miniaturized extraction chromatography: application to isotopic analyses of silicate rocks. *Anal. Chim. Acta* **339**, 79–89 (1997).
19. Kuritani, T. & Nakamura, E. Precise isotope analysis of nanogram-level Pb for natural rock samples without use of double spikes. *Chem. Geol.* **186**, 31–43 (2002).
20. Kuritani, T. & Nakamura, E. Highly precise and accurate isotopic analysis of small amounts of Pb using ^{205}Pb–^{204}Pb and ^{207}Pb–^{204}Pb, two double spikes. *J. Anal. Atom. Spectrom.* **18**, 1464–1470 (2003).
21. Kelley, K. A. & Cottrell, E. Water and the oxidation state of subduction zone magmas. *Science* **325,** 605–607 (2009).
22. Roeder, P. L. & Emslie, R. F. Olivine-liquid equilibrium. *Contrib. Mineral. Petrol.* **29**, 275–289 (1970).
23. Kuritani, T., Yoshida, T., Kimura, J. -I., Takahashi, T., Hirahara, Y., Miyazaki, T., Senda, R., Chang, Q. & Ito, Y. Primary melt from Sannome-gata volcano, NE Japan arc: constraints on generation conditions of rear-arc magmas. *Contrib. Mineral. Petrol.* **167**, 969 (2014).
24. Kuritani, T., Kanai, C., Yamashita, S. & Nakagawa, M. Magma generation conditions at the Akita-Komagatake volcano, Northeast Japan arc: implications

of across-arc variations in mantle melting parameters. *Lithos* **348–349**, 105197 (2019).

25. Kelley, K. A., Plank, T., Grove, T. L., Stolper, E. M., Newman, S. & Hauri, E. Mantle melting as a function of water content beneath back-arc basins. *J. Geophys. Res.* **111**, B09208 (2006).
26. Imai, N., Terashima, S., Itoh, S. & Ando, A. 1994 compilation values for GSJ reference samples, “Igneous rock series”. *Geochem. J.* **29**, 91–95 (1995).
27. Lu, Y.-H., Makishima, A. & Nakamura, E. Coprecipitation of Ti, Mo, Sn and Sb with fluorides and application to determination of B, Ti, Zr, Nb, Mo, Sn, Sb, Hf and Ta by ICPMS. *Chem. Geol.* **236**, 13–26 (2007).
28. Miyazaki, T. & Shuto, K. Sr and Nd isotope ratios of twelve GSJ rock reference samples. *Geochem. J.* **32**, 345–350 (1998).
29. Makishima, A., Nath, B. N. & Nakamura, E. New sequential separation procedure for Sr, Nd and Pb isotope ratio measurement in geological material using MC-ICP-MS and TIMS. *Geochem. J.* **42**, 237-246 (2008).
30. Kuritani, T., Usui, T., Yokoyama, T. & Nakamura, E. Accurate isotopic and concentration analyses of small amounts of Pb using isotope dilution coupled with the double spike technique. *Geostand. Geoanal. Res.* **30**, 209–220 (2006).
